# Decrease in Sperm Parameters in the 21st Century: Obesity, Lifestyle, or Environmental Factors? An Updated Narrative Review

**DOI:** 10.3390/jpm14020198

**Published:** 2024-02-11

**Authors:** Romualdo Sciorio, Luca Tramontano, Mohammed Adel, Steven Fleming

**Affiliations:** 1 Fertility Medicine and Gynaecological Endocrinology Unit, Department Woman-Mother-Child, Lausanne University Hospital, 1011 Lausanne, Switzerland; 2 Department of Women, Infants and Adolescents, Division of Obstetrics, Geneva University Hospitals, 1211 Geneve, Switzerland; luca.tramontano30@gmail.com; 3 Zoology Department, Faculty of Science, Al-Azhar University, Nasr City, Cairo 11651, Egypt; mohamed_ahmed@azhar.edu.eg; 4 Discipline of Anatomy & Histology, School of Medical Sciences, University of Sydney, Sydney, NSW 2006, Australia; blueyfleming@gmail.com

**Keywords:** male infertility, sperm parameters, environmental toxins: bisphenol A and phthalates, obesity, oxidative stress, lifestyle

## Abstract

Semen quality represents a compelling factor for fertility, and delineating the normal values has proven difficult. In the last four decades, several authors have reported a noticeable decline in sperm parameters. Also, studies investigating ‘time to pregnancy’ have shown that fecundity begins to be reduced when sperm numbers decrease below 30 million, even though according to the 6th edition of the WHO manual, the normal value is currently 16 million/mL or 39 million per ejaculate. There exists sufficient data to suggest a decline in sperm counts over time, even though the clear reason for this adverse trend is not well established, but some associations have been hypothesised, such as maternal smoking during pregnancy. Additional potential factors have yet to be fully illustrated but involve poor diet, increased obesity, and exposure to environmental toxins. Moreover, the change in environmental conditions and more common exposure to endocrine-disrupting chemicals (EDCs), such as pesticides and herbicides, as well as bisphenol A, phthalates, polychlorinated biphenyls, and heavy metals, starting from prenatal life and continuing into adulthood, may exhibit probable features explaining the reduction in sperm parameters. Therefore, the main goal of this narrative review is to furnish an overview of the possible effects of exposure to EDCs on testicular function and spermatogenesis and, also, to summarise the evidence regarding a decrease in sperm quality and examine its potential consequences.

## 1. The Global Human Sperm Decline

Several epidemiological studies and the World Health Organization (WHO) manual for human semen assessment have recently reported an unrelenting decline in sperm number by almost half in the last few decades [1,2,3,4,5,6]. The main goal of the WHO manual, which was published for the first time in 1980, was to standardise the protocol for human semen assessment. The manual has been recently updated in 2021, which is the sixth edition. It is also available in several different languages to ensure the same procedures and references for semen analysis independent of the geographical region, to perform consistent diagnosis of male infertility, or to plan infertility treatment for couples in every country in the world. The WHO manual provides standard laboratory methods for semen analysis and the main parameters assessed are sperm volume, the concentration, progressive and total motility, and abnormal forms identified in the morphology. Semen assessments are largely applied worldwide by andrology laboratories to investigate the male reproductive function and to diagnose and plan the best treatment for infertile couples. Briefly, the first manual included only 43 pages, with instructions on semen sample collection and how to perform the assessment of sperm motility, concentration, morphology, and viability. The second edition included additional analyses, such as seminal fluid biochemical tests, the zona-free hamster oocyte penetration test, and “swim-up” as a sperm selection test. Importantly, the second edition introduced “normal” values for sperm concentration (≥20 million/mL) and normal forms (≥50%). The third edition’s main change was the division of the semen assessment into three sections: standard procedures, optional tests, and research tests. The research tests comprised the zona-free hamster oocyte penetration test, human zona pellucida binding tests, the acrosome reaction, and computer-assisted sperm analysis. The third edition also included guidelines and minimal equipment, as well as quality control of the laboratory performance of semen analysis. The fourth manual was launched in 1999, and it reflected advances in the genetics of male infertility, with Y chromosome microdeletions, the introduction of intracytoplasmic sperm injection (ICSI) [7], and the increasing incidence of testicular cancer, as well as declining sperm counts [8]. With the fifth edition, the manual became freely available in print, as well as an electronic version, in order to increase its worldwide distribution. This edition was the most comprehensive edition with 271 pages. Some emphasis was applied to counting errors when an inadequate number of spermatozoa are assessed. Also, semen preparation techniques extended beyond the ejaculate and included the collection of spermatozoa from the testis and epididymis. A chapter on cryopreservation of spermatozoa was also included. Most importantly, the fifth edition for the first time provided reference ranges based on in vivo data derived from studies of fertile men with partners who conceived within 12 months [9]. On 20 July 2021, the WHO launched the sixth edition, following four years of preparation, including interruptions due to the COVID-19 pandemic. It is quite comprehensive and includes information on semen examination and preparation for clinical evaluation, cryopreservation, quality control in the semen analysis, and laboratory assessment to investigate dysfunction in male sexual and reproductive health. The section on the test largely performed during the early days of IVF in the 70s and 80s, examining sperm interaction with cervical mucus, has been eliminated, as the procedure was considered to be no longer used as part of infertility investigation. A normal value for semen concentration was established (≥16 million/mL), as well as normal forms (≥4%). Table 1 summarises the main changes in terms of semen concentration, motility, and normal forms between the last five editions of the WHO manuals. Although the evidence is not unequivocal, male reproductive health seems to be at stake. Indeed, coincident with the temporal decline in male fertility, there has been an increase in cryptorchidism and testicular cancer, which may be explained by exposure to environmental oestrogen-like endocrine disruptors, which appear to be common to both pathologies [10]. Exposure to endocrine disruptors such as oestrogen may also be a causative factor in the temporal decline in testosterone levels that has been observed within men, which would also impact male fertility [10,11,12]. In general, young and adult men do not often require medical consultation for reproductive health issues unless facing difficulties impregnating their partners [13]. Growing data have postulated an association between male subfertility and overall health [14,15]. An investigation from Denmark analysing 4712 men revealed that semen assessment could be considered a biological marker of long-term morbidity and mortality, particularly for cardiovascular diseases and diabetes mellitus [16]. The authors reported a high percentage of hospitalisations, for several illnesses, in men with a low total sperm count and low sperm motility compared to men with normal semen assessment. Among those hospitalised individuals for general health concerns, men with a sperm concentration of 195–200 million/mL were, on average, first admitted to hospital seven years later than their counterparts with a sperm number less than 1 million/mL. The same group reported that the above observations were mainly independent of socioeconomic status and lifestyle factors [17]. These findings were corroborated by a recent review, which demonstrated substantial evidence for a correlation between male general health and sperm quality [1].

## 2. Unhealthy Lifestyles: Smoking and Other Lifestyle Factors

**Figure 1:**
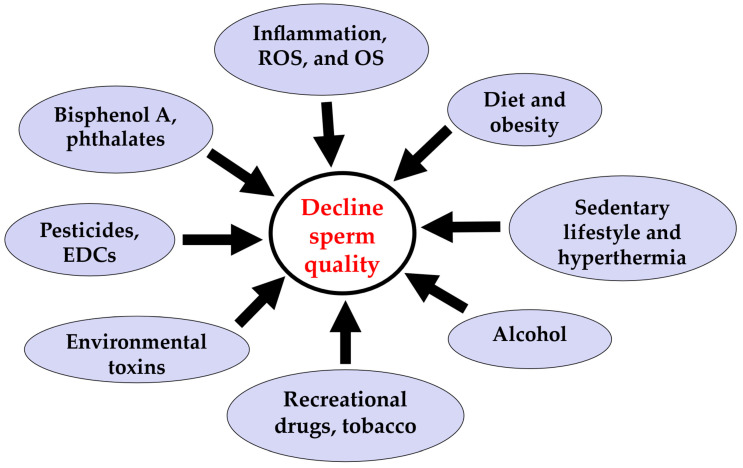
Possible features affecting sperm parameters. EDCs: endocrine-disrupting chemicals; ROS: reactive oxygen species; OS: oxidative stress.

The cause of the worldwide decline in sperm quality is not well understood. Several features seem to influence general health and sperm parameters, including diet and obesity, smoking, alcohol consumption, recreational drug use, pollution, and environmental chemicals and toxins, which will be discussed in this section (Figure 1) [18,19,20,21,22]. Concerns exist that chemical compounds contaminating the environment and radiofrequency radiation pollution might play a role in sperm decline via an adverse effect on the sperm epigenome. Despite its complexity, the epigenome might be vulnerable to environmental conditions [19,20]. Soubry and colleagues also reported that paternal age, environmental pollution, and lifestyle factors, including obesity, have been considered negative factors influencing offspring development and wellbeing [18].

### 2.1. Smoking

Cigarette smoking is identified as a worldwide health problem that contributes to different illnesses and is a cause of premature death. Data suggest that smoking, especially during the preconception time, might negatively impact DNA methylation patterns, thus causing sperm DNA fragmentation and aneuploidy [21,22]. A model to assess the germline age based on sperm DNA methylation at specific loci was developed to estimate the individual’s chronological age with a high degree of accuracy [21]. Moreover, cigarette smoking has been associated with an infertility risk factor and is strongly associated with male sterility [22]. Beal and collaborators analysed the current literature on the subject and found that half of the studies reviewed demonstrated no significant impairment in semen parameters. In contrast, about 40% of the remaining studies found evidence of modest impairment in one or more semen parameters (number, motility, and morphology) when the number of cigarettes smoked per day increased [22]. In addition, the authors reported that paternal smoking during the preconception time was associated with an increased cancer risk in offspring [22]. A study by Sharma and collaborators [23], investigating 5865 men, showed that cigarette smoking is correlated with a decrease in sperm motility and number and that deterioration of sperm parameters is more evident in smoking individuals. Studies have observed that smoking can increase the time for pregnancy: in infertile couples undergoing assisted reproductive treatment (ART), male smoking contributes to a 44% reduction in pregnancy rate after in vitro fertilisation [21]. Smoking exerts its greatest negative impact on genome integrity, where 70% of publications reported some level of smoking-related damage to the genome and epigenome, which might raise the rate of chromosomal aberrations [21,22,23]. A study by Linschooten and colleagues reported that men who smoked during the six months prior to conception were “four times more likely to pass on tandem repeat minisatellite mutations to their children” [24]. Furthermore, the results from meta-analyses provide convincing evidence that paternal preconception smoking significantly raises the risk of cancer in offspring [22]. Most tobacco products contain over 4000 different chemicals and constituents, including nicotine and heavy metals. Between these, cadmium and lead have been individually linked to impaired sperm quality, as has tobacco smoke in general. Smoking appears to reduce sperm concentration, motility, viability, and normal morphology; also, it is associated with DNA damage and leads to the generation of reactive oxygen species (ROS) [25,26]. A study by Pant and co-authors [27] has investigated the correlation between lead and cadmium with sperm quality. They studied fertile and infertile individuals between 20 and 43 years of age. The semen assessment was performed according to the 2010 WHO guidelines. The authors reported that cadmium and lead levels were significantly increased in infertile males. Also, an adverse correlation was found between the seminal concentration of cadmium and lead and sperm numbers, motility, and abnormal forms. One of the main mechanisms involved between smoking and semen impairment seems to be associated with an increase in ROS; cigarette smoking represents a source of pro-oxidants and free radical generators, which can induce oxidative damage and a reduction of redox scavengers in the peripheral blood [28]. Due to this concern, Kiziler and colleagues [29] scrutinised the level of lead and cadmium in seminal plasma and blood, as well as the level of antioxidant defences, in particular, glutathione S-transferase (GST) and reduced glutathione (GSH), in the seminal plasma and spermatozoa from 50 infertile men versus 45 healthy fertile individuals. The authors found that lead and cadmium concentration as well as ROS levels in the smokers’ infertile group were significantly increased compared to the fertile men and the group of nonsmoking infertile males (*p* < 0.001). The levels of GSH and GST activities were reduced in the smoking infertile group as compared to the fertile men and nonsmoking infertile individuals. Finally, sperm parameters of concentration, motility, and morphology in the smokers’ infertile group were observed to be lower than those in the fertile male group and nonsmokers’ infertile group. In this respect, the multifactorial aspects of advanced paternal age (APA) on male reproductive health also need to be considered, such as the accumulation of toxic heavy metals over time [30]. However, another concern greatly studied in male factor infertility is represented by DNA damage in the male germ line, which can be associated with damage to genetic integrity, a reduced fertilisation rate, poor embryo development, and an increased risk of miscarriage [30,31]. Even so, the specific details of the DNA lost integrity are not well elucidated; however, it seems that it is highly associated with DNA compaction during the final stages of spermiogenesis and the damage induced by oxidative stress (OS) [32]. A specific enzyme, 8-oxoguanine DNA glycosylase 1 (OGG1), is involved in the DNA repair pathway in human spermatozoa. Interestingly, it has been elucidated by Smith and collaborators [33] that the activity of this enzyme is significantly reduced when cadmium is present; thus, this heavy metal can be considered as an inhibitor of OGG1 in a time- and dose-dependent manner. However, even though enough scientific evidence suggests that smoking might impair male infertility, more than one-third of male adults worldwide continue to use tobacco, making it perhaps one of the most widespread contributors to declining male fertility [26].

### 2.2. Alcohol

An international cross-sectional study by Jensen and coworkers involving 8344 healthy men from Europe and the USA found only a moderate association between any semen variable and alcohol consumption [34]. However, another investigation reported that teratozoospermia was found in 63% of males who drink alcohol moderately compared to 72% in heavy alcohol-drinking males. None of the males who consumed heavy alcohol had normal sperm parameters. In fact, 64% of them were oligozoospermic, having a lower-than-normal sperm count. Hence, it was hypothesised that the increased testicular impairment is directly associated with day-by-day alcohol consumption. Sen and co-authors [35] analysed the effect that acrylamide in food and alcohol might cause on cell development of the male mouse reproductive system when ingested by the mother during pregnancy and lactation. The authors found that acrylamide and alcohol caused the formation of multinuclear giant cells and degeneration of tubules and maturation-arrested tubules, as well as a reduction in the number of spermatic, Sertoli, and Leydig cells. In addition, lipid peroxidation levels and superoxide dismutase enzyme activity were raised following treatment with acrylamide and alcohol [35]. Similar findings have been observed in human studies [36,37,38,39,40,41] reporting that alcohol might impair male fertility by damaging the anterior pituitary gland, causing the alteration of two fundamental hormones for reproductive function, luteinizing hormone (LH) and follicle-stimulating hormone (FSH), and by interfering with hormone production in the hypothalamus. In the testicles, alcohol might impair the function of Leydig cells, which generate the main male hormone, testosterone. In addition, alcohol can induce dysfunction of the Sertoli cells, which play an essential role in sperm maturation [36]. Also, a reduction in seminal fluid volume and sperm number has been observed in males with high consumption of alcohol, with a reduction in testosterone levels and normal LH, FSH, and prolactin values [40]. With decreased levels of testosterone, the amount of FSH and LH would be expected to rise in order to increase testosterone production. This dysfunction of the pituitary gland to react promptly to the decline in testosterone suggests that alcohol has a critical role in the interaction between the nervous system and the endocrine system [41]. Finally, it is worth mentioning that in alcoholic individuals, many other critical healthy habits are often neglected, such as a requirement for healthy food or physical activity, and sometimes alcohol might be associated with smoking, which renders a more detrimental impairment of male fertility. 

### 2.3. Recreational Drugs

In the last decade, we have witnessed the legalisation of marijuana in different countries; therefore, novel investigations have been published analysing the consequences of marijuana usage on sperm quality. A study by Carroll and collaborators investigated the use of marijuana and its potential effects on semen parameters in individuals undergoing infertility treatments. Following a standard semen assessment conducted on 229 men, the authors concluded that usage of marijuana in both large or moderate amounts had an impairment on sperm morphology and sperm motility [42]. Similar conclusions have also been made by Payne and collaborators in a systematic review [43], confirming that the use of marijuana can be associated with sperm harm, in terms of morphological changes, as well as a reduction in sperm number, motility, and viability. Unfortunately, in modern society, the utilisation of illicit drugs is quite common, especially in Western countries. Those drugs, including cocaine, methamphetamines, or marijuana, might cause detrimental effects on male fertility through impairment of the hypothalamic–pituitary–gonadal (HPG) axis and testicular damage, with alteration in sperm production and function [44]. 

### 2.4. Stress and Poor Sleep

A study by Yuan and coworkers investigated the trend in sperm concentration in 9357 healthy males in the province of Wuhan, Central China. The authors reported that sperm concentration significantly declined with stress over a period of five years [45]. The decline in sperm concentration was more prominent in students versus nonstudents, probably due to a sedentary lifestyle, stress, and lack of sleep. Indeed, there is an association between high work stress and lower sperm concentration and total sperm count. An investigation by Zoe and collaborators [46] found that stress and psychosocial features have a negative effect on sperm quality of semen. Males under serious mental stress had reduced production of testosterone and higher amounts of FSH and LH in comparison to men under normal stress, as well as a reduction in sperm counts, sperm morphology, and motility. The conclusion was that accumulation of work stress is associated with lower sperm parameters, which may have consequences for future reproductive health. Indeed, in modern society, stress is quite common in the form of physical, social, and psychological aspects. In some circumstances, lifestyle factors are also associated with psychological stress including alcohol consumption, cigarette smoking, and an unhealthy diet, which might impair reproductive health [47]. Stress might be differentiated into acute or chronic, which implies the length of the exposure. Acute stress is probably the most frequent form of psychological stress that arises due to demands, tension, and pressure. Repetitive events of acute stress might result in chronic stress, which has been associated with poor pregnancy outcomes following ART cycles [48]. Many authors have assessed and described the impact of mental stress on hormonal changes and their impact on reproductive health [47,48]. Prasad and colleagues established that stress is an important factor that affects the physical and mental health of an individual and might alter the homeostasis of the body [49]. Different consequences might be associated with the body’s different responses, one critical factor being the modification in production and concentrations of various hormones, such as cortisol and prolactin, which may impair sperm parameters. The increased level of stress hormones such as cortisol might have a negative impact on sperm production and quality [47,48,49]. In addition, stress may produce ROS, which further affect male reproduction. The balance between levels of ROS and antioxidants within the testis is critical for reproductive health. ROS impact sperm function, while their accumulation leads to OS and induces apoptosis in germinal cells [49,50,51]. Another feature to mention is the disturbance in sleep patterns that may possibly produce adverse effects on sperm quality and male fertility. A study by Viganò and co-authors analysed the correlation between sleep alteration and sperm quality in about 400 Italian men in couples seeking ART for infertility [52]. They found a total of 46.3% had sleep alterations. Semen volume was reduced in males with difficulty in initiating sleep, while progressive motility was reduced in men with early morning awakening. In obese individuals, semen volume was lower in those men with difficulty in starting sleep. Other studies have reported that limited sleep duration has been implicated as a cause of reduced testosterone levels and fecundability [53,54,55]. Poor sleep quality and duration possibly contribute to abnormal sperm morphology and low sperm concentrations [54]. Interestingly, exposure to light emitted from media devices at night has been shown to impact both the quality of sleep and sperm quality [55]. Finally, to reduce stress levels or sleep disturbance, it is recommended to implement physical activities and a healthy lifestyle, avoiding smoking and use of alcohol, and it is assumed that antioxidants as food supplements could be beneficial to overcome stress-induced OS-mediated deterioration in sperm quality. 

## 3. Environmental Factors and Sperm Quality

Male infertility associated with environmental factors is an emerging feature, and recent studies have investigated them as potential causes of epigenetic dysregulation, with potentially long-term or even transgenerational effects [56,57]. The epigenome is prone to alterations during spermatogenesis and at the early stage of embryo development; at each of these checkpoints, the internal and external environments seem to have a considerable effect on how epigenetics are modulated [18,19,20]. In recent years, some chemical compounds have been extensively investigated to establish any potential association with sperm parameters. Some of the most studied compounds include endocrine-disrupting chemicals (EDCs), such as pesticides, commonly used in agriculture, and plasticisers such as bisphenol A (BPA) and phthalates. 

### 3.1. Endocrine-Disrupting Chemicals

Currently, there is increased attention on the potential effect that EDCs might have on normal homeostatic control and on the reproductive system. Those compounds are commonly found in our food, environment, and consumer products [58]. Essentially, EDCs are compounds that can interfere with any normal activity of the endocrine system, including hormone synthesis, secretion, transport, binding, action, and metabolism. Some EDCs are structurally like steroid hormones, such as androgen and oestrogen, so can mimic their effects via competitive binding to their receptors, leading to reproductive dysfunction via agonist or antagonist effects. For example, they can inhibit the enzymes, 5α-reductase and aromatase, necessary for the conversion of androgens to testosterone and oestrogen, thereby disrupting steroidogenesis and metabolism [59]. Furthermore, modulation of gonadotrophin action within the testis by EDCs may disrupt testosterone production and spermatogenesis. In this respect, LH receptors on Leydig cells mediate the production and secretion of testosterone, while FSH receptors within Sertoli cells mediate their proliferation and initiate spermatogenesis. Some specific pesticides, the so-called organochlorine (OC) pesticides, are widely used all over the world, even if recently banned in some countries, and are extensively utilised in agriculture and the chemical industry. The OCs are fat soluble, can accumulate within adipose tissue, have oestrogenic activity, and are known for their high toxicity, slow degradation, and bioaccumulation [60]. Therefore, APA is a likely risk factor for the accumulation of OCs such as dioxins. A notorious pesticide, banned in the USA but subsequently exported to less developed countries, dibromochloropropane (DBCP), has long been known to result in low levels of FSH due to negative feedback upon the hypothalamic–pituitary axis (HPA) and, consequently, reduced spermatogenesis, though its effects are reversible [61]. A study of 26,400 males exposed to DBCP over a mean period of three years in 12 countries found that 64.3% of them exhibited azoospermia or oligozoospermia [62]. Using the gestating rat model, Sadler-Riggleman and coworkers demonstrated that transient exposure to the well-known pesticides, dichlorodiphenyltrichloroethane (DDT) or vinclozolin, resulted in transgenerational alterations in Sertoli cell DNA methylation, noncoding RNA, and gene expression associated with testis abnormalities [63]. 

### 3.2. Plasticisers

Unfortunately, during the last few decades, it has been recognised that pollution by plastics and plasticisers, virtually everywhere on earth, has had a devastating impact on our environment, from our rivers to our oceans. What is perhaps less well appreciated is their detrimental effect on male fertility via our drinking water and food. Especially, some so-called plasticisers are specific compounds normally added to plastics to increase their flexibility, transparency, and durability or only to improve their manipulation. In this respect, the main culprit appears to be BPA, which is used in polycarbonate plastic food packaging and can linings and can easily be leached from food containers and acquired by any individuals who encounter it [64]. Due to its various endocrine and metabolic disrupting qualities, BPA is regarded as both a xenoestrogen and an obesogen. Preclinical studies demonstrate that BPA inhibits spermatogenesis primarily via its negative impact on testosterone and FSH activity, though clinical studies have tended to be more variable and less conclusive [65]. Notably, in 2011, the European Union banned the inclusion of BPA in baby bottles as a precautionary measure, presumably over concerns regarding the putative increased sensitivity of the neonatal gonad. Exposure to BPA was associated with decreased sperm concentrations and impaired sperm parameters, as well as a raised percentage of immature sperm and reduced testosterone levels [66,67]. Also, phthalates are of particular concern since they are continuously released into the environment, as they are not chemically bound to plastics. The utilisation of phthalates has increased notably in the last few decades, and they can also be found in materials such as cosmetics, paints, and lubricants. Exposure to those chemicals occurs via ingestion or inhalation, or they can be absorbed through the skin. Several studies have reported that constant exposure to phthalates has been associated with an impairment in sperm parameters, including a reduction in sperm number and motility, as well as an increase in abnormal forms and DNA damage [68,69,70,71,72]. Phthalates are present within urinary metabolites, with significantly higher levels being found within infertile men, and have been correlated with the downregulation of testosterone and insulin-like factor 3, which is a marker of Leydig cell function [69]. Increased urinary BPA concentrations have also been associated with reduced sperm parameters and increased sperm DNA damage [72,73,74,75]. Indeed, different population studies performed on the effect of BPA conjugates have reported a value to be over the safety limit in 90% of individuals investigated, and this might represent an alarming feature considering that BPA conjugates could disrupt endocrine function since they can bind to steroid receptors [71,72,73]. In both instances, any shift in the testosterone/oestrogen ratio would further downregulate testosterone secretion and Sertoli cell function via negative feedback by oestradiol upon hypophyseal LH and FSH secretion, respectively, thereby leading to reduced spermatogenesis [74,75,76,77,78,79].

### 3.3. Effect of Endocrine Disruptors

In humans, hormones are extremely important and have a central role in cell growth and differentiation. In males, spermatogenesis, which is the production of functional spermatozoa, as well as the synthesis of testosterone, the main male sex hormone, are both regulated by the hypothalamus and the anterior pituitary gland present within the human brain. A negative feedback mechanism on hormone synthesis is controlled by testosterone and its metabolites, oestradiol and dihydrotestosterone, as well as via inhibin B feedback from the testis upon hypothalamic gonadotropin-releasing hormone (GnRH) and pituitary gonadotropin discharge [80]. Endocrine disruptors can affect both the HPA and sperm function, altering sperm parameters [23,81,82,83]. Conditions that affect the HPA will eventually affect GnRH and hence the levels of FSH, LH, and prolactin. These conditions include Kallmann syndrome (isolated gonadotropin deficiency), hyperprolactinemia, and hypopituitarism. Hyperprolactinemia may be due to diseases affecting the hypothalamus and pituitary gland or secondary to diseases of other organs such as the liver, kidneys, and thyroid [84]. Hyperprolactinemia may cause hypogonadism, erectile dysfunction, decreased libido, gynecomastia, and infertility. Disruption of the HPG axis might cause a significant reduction in the production of testosterone. Indeed, it is well documented that a high level of testosterone is critical for testicular development in prepubertal males and for the maintenance of masculinity in adulthood. Compared to serum, the intratesticular testosterone level is about 100 times higher, highlighting the importance of the blood–testis barrier (BTB), composed of specific cell junctions and adhesion between Sertoli cells and germ cells [85]. 

## 4. Air Pollution

**Figure 2:**
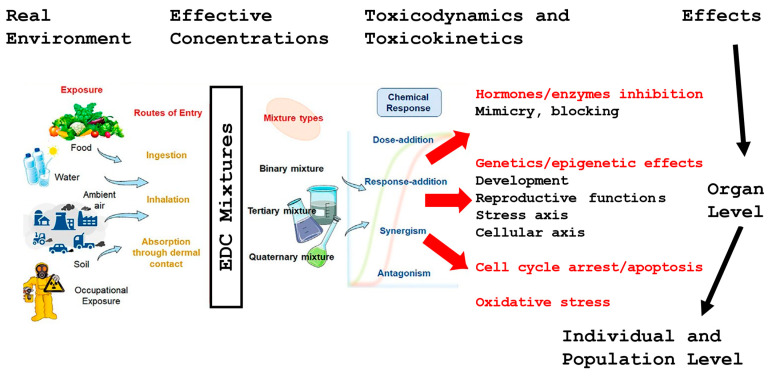
Lifestyle factors affecting human fertility. Adapted from Dutta and colleagues [95].

Environmental pollution has been correlated with a reduction in sperm quality, especially when analysing sperm morphology (Figure 2) [86]. Ample publications have provided sufficient evidence on the detrimental effects of air pollution on sperm parameters, as well as volatile organic solvents, silicones, chemical dust, and pesticides on male fertility and fertility complications [82,83,86,87,88,89,90]. Experimental studies have identified the presence of several chemical contaminants in the urine or blood, with detrimental effects on semen parameters [86,87,88,89,90]. Air contamination includes increments of nitrous dioxide (NO_2_), sulphur dioxide (SO_2_), carbon monoxide (CO), and carbon dioxide (CO_2_), as well as ozone and lead. Particulate matter (PM) in the respirable range (PM 2.5 μm, 5.0 μm, and 10 μm) is notably harmful, since it can bring to the bloodstream multiple trace elements and polycyclic aromatic hydrocarbons, a group of compounds that includes several endocrine disruptors. Rubes and collaborators [91] showed that air pollution was associated with increased DNA fragmentation in human sperm without other changes related to semen quality. These results confirmed previous evidence of sperm morphological abnormalities [88,92] in male residents in a suburb with high levels of air pollution in the Czech Republic [93]. Additional investigation by Radwan and coworkers [94] analysing infertile men with normal semen concentration found a positive correlation between poor sperm morphology and exposure to all examined air pollutants.

## 5. Diet, Sedentary Lifestyle, and Hyperthermia

As discussed previously, many factors influence sperm quality, among which lifestyle, sport, and physical activities, as well as diet, have been objects of investigation by several research groups. In particular, the Mediterranean diet, which basically involves a large number of fruits, legumes, vegetables, grains, fish, and the use of extra-virgin olive oil, associated with reasonable physical activity, has been correlated with good semen quality [96,97]. The benefit of the Mediterranean diet has been principally demonstrated to be the result of a decreased risk of cardiovascular disorders, which seems to be due to its antioxidant and anti-inflammatory characteristics [98]. A randomised controlled study performed by Montano and co-authors [99] investigated the effect that diet and physical activities might have on semen parameters in 263 young males living in Italy. The authors found that a Mediterranean diet and physical activity induced an increase in sperm number, total motility, and better morphology. It was also reported that a sedentary lifestyle with more than four hours of sitting per day was significantly associated with a higher percentage of immotile spermatozoa [99]. On the other hand, studies have indicated that regular overconsumption of prepared meat, fatty dairy products, and sweet drinks by men is likely to result in poor semen quality. Associated significant health issues include testicular cancer, urogenital malignancies, diabetes mellitus, metabolic disorders, and cardiovascular disease [99,100]. Another critical factor that needs to be mentioned is prolonged exposure to scrotal hyperthermia and its impairment of sperm quality. A recent study by Budzinska and colleagues [101] analysed this aspect, as well as oxidative and apoptotic markers in four groups of males: men with varicocele (n = 78), drivers (n = 54), infertile individuals who were not in contact with genital heat stress (n = 37), and fertile men as controls (n = 29). The results showed that thermal alteration induces a substantial deterioration in standard semen assessment. Also, a strongly apoptotic phenotype was found in sperm, with increased DNA fragmentation and reduced mitochondrial membrane potential [101]. In addition, those groups which were exposed to genital heat stress revealed a clearly increased production of superoxide anion in the mitochondrial chain. This allowed the authors to conclude that exposure of the testis to hyperthermia in real-life scenarios is sufficient to induce a decline in human sperm parameters due to harm caused by the OS cascade in ejaculated sperm, affecting plasma membrane fluidity, mitochondrial homeostasis, and sperm DNA integrity. Another study by the same group [102] focused on the importance of hyperthermia and impairment of sperm quality. In particular, they reported modifications in the seminal oxidative system, the level of DNA integrity, and inflammatory factors in professional groups exposed to long-term heating stress.

### 5.1. Obesity

The worldwide incidence of obesity has increased significantly since 1975, and several studies have shown that overweight and obesity can cause male infertility [103,104,105,106,107,108,109]. Indeed, overweight and obesity is one of the major contributory factors associated with the reduction in sperm number and low total sperm concentration, frequently observed in obese or overweight males [107,108,109]. The mechanisms believed to be responsible for this include hypogonadism due to aromatisation of testosterone into oestrogen, impairment of Sertoli cell function due to decreased inhibin B/FSH ratio, elevated scrotal temperature due to lower abdominal obesity, and accumulation of toxins within adipose tissue [110]. In this respect, adipose tissue is effectively an endocrine-active organ. The excess of adipocyte cells and white adipose tissue in overweight or obese individuals prolongs the transformation of cholesterol to testosterone, impacting the HPG axis and reducing gonadotropin release with negative effects on sperm production [105]. Furthermore, obesity leads to neuroendocrine dysfunction due to leptin release impacting kisspeptin neurons, which in turn interact directly with LH and GnRH receptors [111]. Alarmingly, studies have found that paternal obesity is positively associated with increased body fat in prepubertal offspring [106] and effects upon the methylation function of specific loci of imprinting genes in offspring [18]. A study by Lin and colleagues performed in China on about 30,000 males investigated the correlation between the paternal body mass index (BMI) at the time of pregnancy and neonatal outcomes and long-term prognosis in offspring. The authors found a raised percentage of hypertensive disorders during pregnancy, caesarean delivery, and gestational weight gain, as well as obesity, in those adolescents born from obese or overweight males at the time of pregnancy [106]. An investigation performed by Soubry and coworkers revealed an alteration in methylation function; in particular, a reduction in methylation of *MEST, PEG3*, and *NNAT* genes was observed in those babies conceived from obese males compared with babies born to nonobese parents [104].

### 5.2. Metabolic Syndrome and Its Effect upon Sperm Parameters

Studies have reported a correlation between metabolic syndrome and sperm parameters, especially for idiopathic infertility in males [108,109]. Metabolic syndrome is established when three of the following features are present: increased waist circumference, arterial blood pressure or fasting glucose, hypertension, high triglycerides, and reduced high-density lipoprotein cholesterol. Males with a higher number of metabolic syndrome elements had an increased risk of producing sperm with high abnormal forms (poor morphology) and are associated with a diminished percentage of motile spermatozoa. Hypertension, increased waist circumference, and increased serum glucose were associated with a decreased percentage of normal sperm morphology. This concern has been investigated by Chen and collaborators [109], who analysed the association between metabolic syndrome and sperm quality in about 8000 males in Taiwan who underwent private medical assessment. The authors reported a significant association between metabolic syndrome, especially blood pressure, serum glucose, and waist circumference, and a diminished percentage of normal sperm morphology and reduced motility. Furthermore, both human and animal studies have reported the correlation between male obesity and reduced sperm parameters [105,106,107,108,109]. Studies have shown that abnormal semen parameters can be ascribed to overweight and obesity, such as reduced sperm number, progressive motility, and elevated abnormal sperm morphology [109,112]. Similar findings have been observed in animal studies where obesity was induced artificially by diet, consequently resulting in male subfertility [112,113]. A paper by Meng and collaborators [114] analysed the correlation between paternal BMI and neonatal outcomes of singletons born following frozen-thawed embryo transfer treatments. The authors analysed about 8000 singleton deliveries according to four groups of paternal BMIs: paternal underweight, normal weight, overweight, and obesity. The authors found that the percentage of large for gestational age babies was increased in the groups of paternal obesity and overweight compared to the underweight group. However, several other altered factors may impair sperm quality including sex hormone imbalance, OS, and chronic inflammation. Notably, there is also some evidence indicating that weight loss, by exercise, lifestyle changes, or bariatric surgery, can efficiently result in increased serum testosterone levels and sperm counts [115], suggesting possible benefits for weight loss on male fertility [116]. Furthermore, an alteration in the acrosome reaction (AR) in overweight and obese males has also been described [117]. Although the association between male obesity and sperm AR is still an object of debate, it is reasonable to assume that the impact of obesity on spermatogenesis and sperm maturation, which results in OS and membranous lipid alteration, may also cause some defects in the AR. Comparative studies have identified the expressed proteins in semen samples from obese males and, using differential gel electrophoresis or liquid chromatography–tandem mass spectrometry, have observed less abundant proteins in obesity-associated asthenozoospermia. The biological functions of these proteins include actin organisation, flagellar assembly, vesicular traffic, protein degradation, and stress resistance, as well as involvement in acrosome biogenesis, nuclear reshaping, and flagellum formation during spermiogenesis, the depletion of which may directly cause abnormal sperm function [118,119].

### 5.3. Obesity and Inflammation Processes

Accumulated evidence from the literature reported an association between chronic inflammation or the proinflammatory state and obese or overweight men [120,121,122]. White adipocytes produce several molecules, some of these compounds including interleukins (IL-1, IL-6 and IL-18) or tumour necrosis factor-α (TNF-α), defined as proinflammatory cytokines, which are mediators of the inflammation process and attract macrophages. In animal models, it has been well known that proinflammatory cytokines commit tissues to alterations in glucose homeostasis and insulin resistance that are often linked with obesity. In addition to the adipocytes, these proinflammatory cytokines, such as TNF-α and IL-6, are also increased in the serum, testicular tissue, and the seminal plasma of mice [123,124]. It is reported that proinflammatory cytokines exert some impacts on the HPG axis and fertility [125]. Systemic inflammatory diseases, such as rheumatoid arthritis, might cause a reduction in the production of testosterone [124]. The proinflammatory cytokine TNF-α directly inhibits LH function and, subsequently, leads to low testosterone levels and male subfertility [126]. Therefore, increased systemic inflammatory cytokines in the serum of obese or overweight men can cause a reduction in androgen production at various levels of the hypothalamic–pituitary–Leydig cell axis. In the testis, proinflammatory cytokines can directly impair the seminiferous epithelium. Sertoli cells respond to many of these proinflammatory cytokines, most notably IL-1, TNF-α, and interferon. These molecules affect the expression and assembly of the junctional proteins, zonulin/zonula occludens-1 (ZO-1), occludin, claudins, and actin–myosin cytoskeletal proteins; thus, they might cause openings of cell junctions between adjacent Sertoli cells, impairing the seminiferous epithelium and sperm production [127,128,129]. Damage to the BTB and decreased expression of junctional proteins in Sertoli cells has been described in many diet-induced obese animal models [114]. Additionally, sperm maturation in the epididymis is crucial for sperm to acquire motility and fertility. The epididymal epithelium transports proteins and lipids through epididymosomes to the sperm membrane, which is necessary for sperm maturation [130]. Proinflammatory conditions induced by obesity can also damage epididymal epithelium function by altering the environment within the epididymis, modifying epididymosome content, and increasing the influx of neutrophils and macrophages to the epididymal lumen, resulting in higher cytokine expression and epithelial apoptosis, thus impeding sperm maturation and fertilisation ability [130]. Taken together, proinflammatory cytokines produced within the testis and epididymis, or originating from the circulation during systemic inflammation, might infringe on and disturb the critical processes of regulation of spermatogenesis and sperm maturation. 

## 6. Effect of Radiofrequency Radiation on Sperm Parameters 

In the last twenty years, we have witnessed a sharp increase in the use and application of mobile phones in our lives, which has contributed to radiofrequency electromagnetic radiation (RF-EMR) environmental pollution. Consequently, the level of RF-EMR has increased in public areas, schools, and at our houses, increasing concern in the scientific community about possible adverse effects on human health, particularly on the male reproductive system. This concern has been investigated by several authors [131,132,133,134,135]. Human testicles seem to be susceptible to RF-EMR, and it seems that one of the main impairments is induced by increased testicular temperature, debilitating sperm quality [134,135,136,137]. Testicles are very sensitive organs and exposure to these types of radiation might impair both germinal cell precursors of spermatozoa and mature spermatozoa [136]. The specific process of this damage is not fully understood; however, it seems dependent upon the so-called “thermal effect”, which might disrupt cell function and development [138,139,140]. The potential harm of heating stress has also been reported on germ cells, which have high mitotic activity, and on spermatocytes and early round spermatids, which are vulnerable to temperature alteration [141,142,143,144,145]. In animal models, the evidence is more clear, since various authors have shown that a rise in testicular temperature by exposure to RF-EMR directly affects the seminiferous tubular epithelium, as well as histological alteration, and causes semen abnormalities with decreased sperm count and normal morphology [145,146,147]. Additionally, adverse consequences have been described in germ cells, including damage caused by apoptosis, autophagy, and OS [146,147,148,149,150]. Prausnitz and Susskind in 1962 [151] reported for the first time the impairment that microwave radiation may induce in the testes. More recently, Agarwal and colleagues [134] investigated the relationship between cell phone usage and semen parameters, and they found an association between the usage of telephones and a decrease in sperm number, motility, and morphology. The decrease was associated with the duration of daily exposure to cell phones and was found to be independent of the initial semen quality. This conclusion has been reinforced by several authors [133,134,135,138,139]. However, the published literature remains equivocal concerning the effect of mobile phone use on sperm parameters, with studies reporting decreased sperm motility, sperm concentration, and poor morphology [132,133,134,135,138,139,140], whereas others have shown no apparent effect on sperm quality and quantity [152,153], advocating the need for additional studies to clarify this aspect.

## 7. Oxidative Stress and Sperm Quality

**Figure 3:**
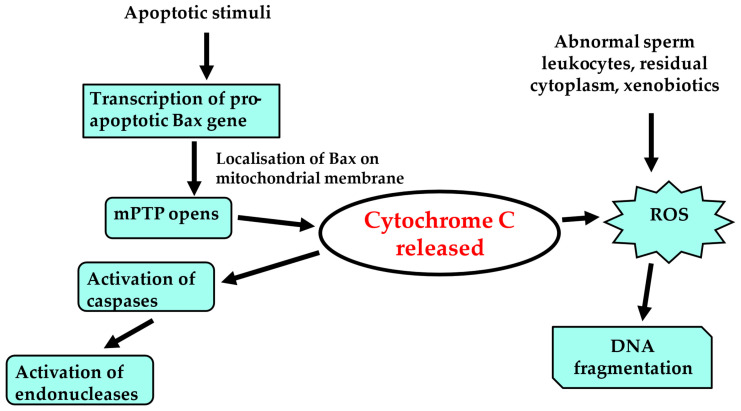
Concurrent pathways believed to be involved in RF-EMR, ROS, and sperm DNA damage/fragmentation. Adapted from Thomson et al., 2009 [160]. RF-EMR: radiofrequency electromagnetic radiation; ROS: reactive oxygen species; DNA: deoxyribonucleic acid; mPTP: mitochondrial permeability transition pore.

Recent investigations seem to indicate that the main testicular harm might be correlated to increased production of ROS and OS (Figure 3), leading to changes in protein conformation and induction of DNA damage [154,155,156,157]. Increased OS is a condition in which the natural balance between oxidants and antioxidants is disturbed; thus, free radicals are produced containing an uneven number of electrons, which react with other molecules to cause a series of chemical reactions that might be toxic to cells, tissues, and organs, including gametes and embryos [155,156,157,158]. The effects caused by mobile phones are associated with increased production of seminal ROS and reduction in antioxidant enzymes, chromosomal abnormalities, micronuclei formation, and changes in the sperm membrane potential, as well as increased apoptosis and DNA fragmentation [158,159,160,161,162,163]. Sperm DNA damage induced by OS is marked by the generation of the DNA base adduct, 8-hydroxy-2′-deoxyguanosine (8OHdG), the only repair mechanism available to spermatozoa being OGG-1, which is the first enzyme in the base excision repair (BER) pathway [32]. Depending upon the extent of single or double-strand breaks in the sperm DNA and the ability of the oocyte to repair them at fertilisation, there can be differential impacts upon embryogenesis, resulting in an increased risk of implantation failure or miscarriage [164]. Leydig cells, seminiferous tubules, and spermatozoa are the main targets of the damage induced by OS caused by the application of cell phones. The impairments might also repress testicular steroidogenesis and reduce testosterone levels, with disturbances in spermatogenesis [157,158,159,160,161,162]. A study by Desai and colleagues [165] demonstrated that RF-EMR exposure causes an increase in OS able to induce sperm DNA damage and stimulate sperm cell death. An association has also been found between OS, enhanced lipid peroxidation, and changes in the body’s antioxidant functions; the sperm plasmalemma is particularly susceptible to lipid peroxidation due to its high polyunsaturated fatty acid content and the formation of the toxic lipid peroxidation products, 4-hydroxynonenal (4-HNE), malondialdehyde (MDA), and acrolein. These byproducts of lipid peroxidation disrupt the sperm plasmalemma and mitochondrial proteins of the electron transport chain, thereby decreasing sperm motility and potentially harming fertilisation potential [166]. Indeed, sperm motility and sperm-oocyte recognition are particularly sensitive to OS due to loss of membrane fluidity and integrity [167]. Furthermore, it is well known that mitochondrial dysfunction negatively affects sperm motility and increases abnormal forms [168]. Mitochondria are fundamental organelles critically important in providing energy for sperm motility; any metabolic disruption in the electron transport chain can significantly increase mitochondrial ROS production, affecting sperm quality [134,168]. Mobile phone exposure can increase mitochondrial ROS production, induce sperm DNA fragmentation, and decrease sperm motility and viability [169,170]. Excessive ROS can alter the function of several seminal enzymes, including superoxide dismutase (SOD), catalase (CAT), and glutathione peroxidase (GPx), which play an important part in the protection of spermatozoa from attack by ROS. It has been reported that a reduction in glutathione and superoxide production after exposure to mobile phone radiation is responsible for the damage to sperm membranes because of OS and increased ROS production [171].

## 8. Conclusive Remarks

The question of declining male fertility remains an open debate and is not yet conclusive. However, sperm count and fertility are not synonymous, and a reduction in sperm parameters does not inevitably mean a decline in male fertility; thus, the association between reduced sperm count and motility and male fertility still needs to be fully clarified. In addition, according to the WHO, the results of all tests conducted on both partners should be considered in the holistic treatment of the couple. Nevertheless, the integrity of the sperm genome is indicated as the most reliable biomarker for causes of male infertility. In this review paper, several features that might be potentially associated with the decline in sperm parameters have been discussed, such as diet, obesity, the inflammation process, and exposure to environmental toxins. Also, the impact of BPA or phthalates was evaluated, including exposure to endocrine disruptors, which by several authors have been reported to compromise testicular function in adulthood and induce dysfunction, malformations, and, finally, induce male infertility. Probably, there are a multitude of reasons to account for the decline in sperm quality. It is important to mention that most investigations have been conducted on animal models due to the ethical limitations of completing such experiments in humans. Finally, we surely believe that there is an imperative need for more prospective and large studies with the goal of giving definite answers to the topic of declining sperm counts.

## Figures and Tables

**Table 1:** Depicts the main changes in the last 5 editions and the main semen parameters analysed. * Morphology using Kruger’s strict criteria [3].

WHO Edition	Volume (mL)	Sperm Concentration (10^6^/mL)/Total Number (10^6^/Ejaculate)	Total/Progressive Sperm Motility (%)	Normal Forms (%)
2nd	≥2	≥20/≥40	≥50/≥25	≥50
3rd	≥2	≥20/≥40	≥50/≥25	≥30
4th	≥2	≥20/≥40	≥50/≥25	Normal forms <15 might be associated with decreased in vitro fertilisation success
5th	≥1.5	≥15/≥39	≥40/≥32	≥4 *
6th	≥1.4	≥16/≥39	≥42/≥30	≥4 *

## Data Availability

No data are available.
